# Smart Devices–Based Longitudinal Assessment of Patterns of Insomnia (SLEEPI): Protocol for Large-Scale, Digital, Longitudinal, and Decentralized Clinical Trials

**DOI:** 10.2196/92341

**Published:** 2026-11-02

**Authors:** Marie-Ange Stefanos, Martin de Gourcuff, Guillaume de Laboulaye, David Campo, Pierre Alexis Geoffroy, Anna Sigridur Islind

**Affiliations:** 1 Inserm, F-75019, NeuroDiderot, SleepCmd, Université Paris Cité, Bingen, 48 boulevard Sérurier, Paris, Île-de-France, 75019, France; 2 Department of Computer Science, Reykjavík University, Reykjavik, Iceland; 3 Withings (France), 2 rue Maurice Hartmann, Issy-les-Moulineaux, Île-de-France, 92130, France, 33 646668445; 4 Hopital Bichat - Claude Bernard, F-75018, Département de psychiatrie et d’addictologie, AP-HP, GHU Paris Nord, DMU Neurosciences, Paris, France; 5 GHU Paris - Psychiatry & Neurosciences, Centre ChronoS, Paris, France

**Keywords:** decentralized trials, large-scale clinical trials, insomnia, digital protocol, longitudinal data collection, wearables, nearable devices, continuous monitoring, digital questionnaires

## Abstract

**Background:**

Personalized medicine requires an understanding of health conditions in real-world settings, but traditional clinical tools are often poorly suited for longitudinal tracking due to logistical hurdles, high participant burden, and data quality issues. While consumer wearables offer a scalable alternative, decentralized clinical trials introduce new challenges in patient recruitment, sustained engagement, and data management. Insomnia serves as a suitable use case for this protocol due to its global prevalence, frequent underdiagnosis, and the well-documented discrepancy between subjective patient reports and objective physiological metrics.

**Objective:**

We present a research protocol for large-scale decentralized clinical trials and report its recruitment performance, applied to a longitudinal study on insomnia, called the SLEEPI (Smart Devices–Based Longitudinal Assessment of Patterns of Insomnia) study, using an established base of commercial devices with limited additional manual intervention. This study aims to develop a machine learning model classifying subjective insomnia presence at the participant level against *DSM-5*-TR (*Diagnostic and Statistical Manual of Mental Disorders*, Fifth Edition, Text Revision) criteria.

**Methods:**

Two data streams are collected: subjective, clinically anchored patient reports and objective physiological metrics (eg, heart and respiratory rates) captured via unobtrusive wearable and nearable sensors. This study is executed through a digital protocol designed to conduct a large-scale, longitudinal clinical trial. This protocol replaces the manual interventions of site-based studies with digital tools that manage the full participation process. We detail this protocol from initial recruitment through engagement, adherence, study monitoring, and data management to study completion or withdrawal. The data collected are intended to train and evaluate a machine learning model for subjective insomnia classification. The recruitment objectives were determined by using simulations to determine the minimal sample size for that purpose and by applying a prospective attrition model to calculate the initial contact volume needed to reach that targeted sample.

**Results:**

This protocol enables the continuous monitoring of over 10,000 individuals for 26 weeks. Enrollment metrics exceeded projections: initial survey response rates reached 16% (vs 10% projected), the consent rate was 96% (vs 77% projected), and the final inclusion rate was 90% (vs 81% projected). Recruitment opened in February 2025, and the initial wave closed 6 weeks later with 10,823 individuals included. Data collection is projected to end in June 2027. Preliminary analyses have been conducted, and the first results are expected in the summer of 2027.

**Conclusions:**

This protocol uses preexisting user bases of connected devices to conduct the trial with limited additional manual intervention. Beyond insomnia, the protocol is intended as a model for generating large-scale, real-world clinical datasets, presenting new opportunities for research in other complex areas such as immunology, mental health, and cardiovascular diseases.

## Introduction

Insomnia is a prevalent chronic sleep disorder characterized by difficulties in initiating or maintaining sleep, leading to significant daytime impairments such as fatigue and concentration difficulties [1,2]. The disorder affects approximately 30% of the world population [3-5], yet it remains frequently underdiagnosed, with fewer than 15% of affected individuals seeking medical intervention [6]. One challenge in its characterization is the notable discrepancy between patients’ subjective reports and objective metrics obtained using standard assessment tools [7,8]. Although diagnosis currently relies on clinical evaluation of subjective patient reports, it could be augmented and refined by objective, longitudinal data analysis [6,9].

Traditional data collection devices have well-documented limitations that impede the application of data science techniques to clinical research on insomnia: actigraphy can erroneously interpret prolonged wakeful immobility as sleep [10], while polysomnography is resource-intensive, obtrusive, and is generally limited to 1 or 2 nights, which is an inadequate time frame for capturing the natural variability of sleep patterns over time [11,12]. The datasets collected with these instruments are not only inadequate for training machine learning (ML) models but also present methodological issues. Despite European guidelines recommending large sample sizes [1], studies frequently include only up to 100 participants [13,14], which is insufficient to train ML models that can generalize across diverse demographic and clinical subgroups within the study sample, such as sex, age, BMI, race, and socioeconomic status [15]. Moreover, data collection periods are often too brief, typically lasting 1 to 8 nights [16-21], which falls short of the 14 nights to 2 months recommended to capture the disorder’s variability [22]. Furthermore, these datasets usually lack demographic diversity (eg, cohorts consisting only of male participants [23]) and rely on data from artificial laboratory settings [24,25] that fail to reflect real-world patient conditions.

Our central hypothesis is that ML models, when trained on high-dimensional and longitudinal sensor data, can identify objective physiological signatures predictive of a patient’s subjective experience of insomnia, thereby elucidating complex, nonlinear relationships that traditional methods such as actigraphy fail to capture.

Testing this hypothesis requires longitudinal data from a population large and diverse enough to train such models, which prior insomnia studies have not produced. Conducting large-scale, decentralized, and longitudinal clinical studies in real-world settings is essential for a better understanding of health conditions [26], yet such research faces significant logistical and methodological challenges, including recruitment bottlenecks, participant burden, and data quality assurance [27]. Building on previous decentralized trial models [28-30], we propose a scalable protocol that uses an established base of commercial smart devices. The protocol reduces operational demands through 5 components: recruitment from a preexisting user base, passive data collection, automated data management, a structured withdrawal process, and a model forecasting participant dropout at each stage of the study, which supports resource planning. We developed this new methodology for a decentralized clinical trial (DCT) on insomnia, a field in which decentralized methods remain uncommon.

To address these clinical and research gaps, we designed the SLEEPI (Smart Devices–Based Longitudinal Assessment of Patterns of Insomnia) study. This large-scale, DCT captures real-world data by monitoring each participant in their homes with connected wearable and nearable sensors for a 26-week period in order to overcome the limitations of prior research by capturing the episodic nature and variability of insomnia characteristics, such as initial vs terminal insomnia. While this type of dataset has biases, it has the benefit of capturing the demographic and clinical heterogeneity characteristic of real-world populations, which is often absent in the highly-selected cohorts of traditional laboratory studies.

To test our hypothesis that ML models can identify new physiological signatures of subjective insomnia, our methodology captures 2 synchronized data streams: first, subjective data in the form of clinically-anchored patient-reported outcomes (PROs) from a mobile app, which serve as the ground truth; and second, objective data from the wearable and nearable devices described below. Previous DCTs have demonstrated the feasibility of using consumer-grade devices to create extensive datasets [28,29,31] from which phenotyping of complex disorders into homogeneous subgroups is possible [12,32-35]. In our study, the devices’ embedded sensors, photoplethysmography, accelerometers, microphones, and pressure and temperature sensors enable the passive, unobtrusive, and continuous collection of vital parameters such as heart rate, respiratory rate, and variables related to activity and sleep [22,36]. Together, these 2 streams provide the labeled dataset required to train and evaluate a model classifying the presence of subjective insomnia.

## Methods

### Ethical Considerations

The SLEEPI study was approved by CPP (Comités de Protection des Personnes) Ouest III, an independent French ethics committee, on July 25, 2024, under ID number 2024-A01028-39. Informed consent was obtained from all participants involved in the study. Participants could withdraw their consent at any time. All data are pseudonymized and processed in compliance with the General Data Protection Regulation (GDPR) [37] and applicable French data protection laws [38], as detailed in the *Data Security and Regulatory Compliance* section. The participant compensation structure was approved by the same committee.

### Study Design

The primary end point of the SLEEPI study is the performance of a ML model in classifying the presence of insomnia at the participant level, against the subjective criteria for insomnia diagnosis of the *DSM-5*-TR (*Diagnostic and Statistical Manual of Mental Disorders*, Fifth Edition, Text Revision), reported as accuracy, sensitivity, and specificity on the analysis set defined in Multimedia Appendix 1. The secondary end point is participant adherence, measured as the proportion of participants meeting both data-contribution criteria.

The identification of physiological signatures follows from interpreting models trained for the primary end point and is not a separate end point. The classification of insomnia severity, derived from the Insomnia Severity Index (ISI), and the analysis of the data’s temporal structure are exploratory.

To achieve these goals, the SLEEPI study involves 3 Withings sleep-related devices: the nearable Withings Sleep Analyzer (WSA) and the wearable smartwatches ScanWatch (SW) and ScanWatch 2 (SW2). Together, these devices continuously record objective data to capture physiological and behavioral parameters: detailed sleep architecture, activity intensity, temperature variations, and a range of cardiorespiratory variables such as heart rate, heart rate variability, oxygen saturation, and snoring duration [36,39-44]. The dataset used to train the ML models exclusively contains physiological metrics computed from raw sensor data. The unprocessed source signals, such as acceleration and photoplethysmograph, are not included. Complementing these objective measurements, subjective data are gathered through digital questionnaires administered via the Withings mobile app (WMA). These surveys include questions for insomnia diagnosis based on *DSM-5*-TR criteria and clinically validated scales to quantify symptom severity: the ISI [45], the Patient Health Questionnaire-9 (PHQ-9) [46], and the Generalized Anxiety Disorder-7 (GAD-7) [47]. The questionnaires also collect demographic, clinical, and environmental data for the following purposes: (1) implementing screening procedures to limit low-quality data, (2) allowing for subgroup analyses, (3) identifying at-risk populations, and (4) adjusting for confounding variables.

Following the collection of informed consent and verification that all inclusion criteria are met, each participant begins a 26-week monitoring period under free-living conditions, during which they are required to use at least one of the study devices (WSA, SW, or SW2) and complete several questionnaires sent via the WMA. The questionnaire timeline is shown in Figure 1. Once the monitoring period ends, compensation is provided 8 weeks thereafter to participants who have met predefined criteria for data contribution, while individual results are shared with all participants 16 weeks later.

**Figure 1.**
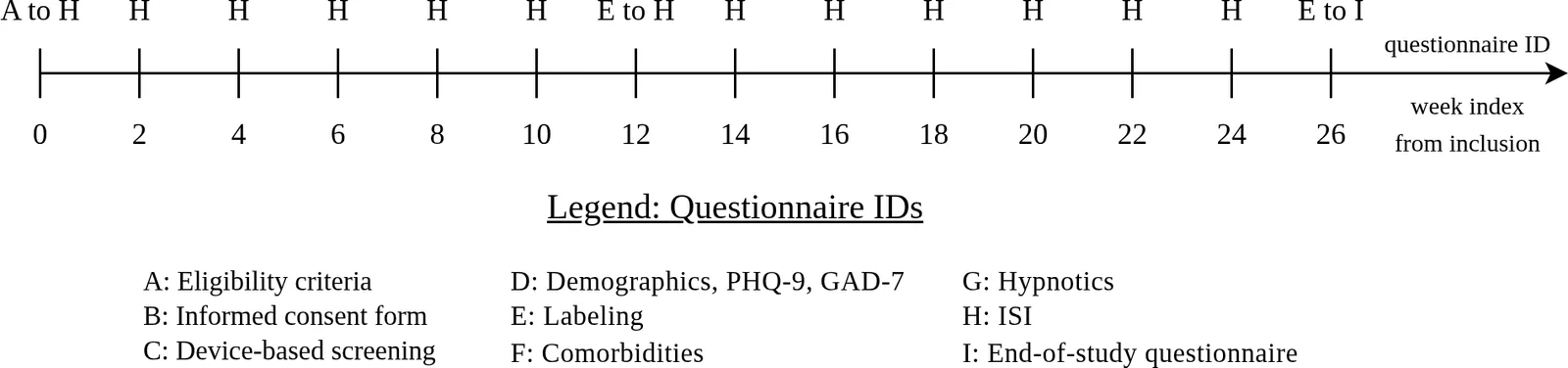
Timing and sequence of study questionnaire administrations during the monitoring phase. GAD-7: Generalized Anxiety Disorder-7 [47]; ISI: Insomnia Severity Index [45]; PHQ-9: Patient Health Questionnaire-9 [46].

### PRO-Derived Reference Labels for Physiological Signature Identification

The dataset created has several challenging characteristics: it is large-scale, multidimensional, longitudinal, and inherently sparse due to highly irregular sampling frequencies that vary at multiple levels—between individuals, across different data dimensions, and even over time for a single variable. This overall complexity renders simple signal processing and rule-based approaches ineffective for reliably identifying subjective insomnia. Therefore, we will adopt a supervised ML approach to automatically discover predictive patterns within the data. This approach addresses a binary classification task that differentiates individuals with and without subjective insomnia and, as an exploratory extension, classifies insomnia severity into classes derived from the ISI. The inputs for these models are passively collected sensor data recorded over each participant’s monitoring period.

For each of these tasks, a supervised ML model requires reference labels for each individual—that is, the subjective insomnia status assigned to that individual—which the model learns to predict from the input data. These labels will be derived from PROs obtained through the questionnaires, and therefore record the participant’s own report; no clinical assessment contributes to the label. The items implementing the diagnostic criteria from the *DSM-5*-TR were written for this study so that they can be self-administered at scale, whereas the severity questionnaire (ISI) is used as published. To distinguish between individuals with and without subjective insomnia, the label is built on the subjective criteria for the diagnosis of insomnia from the *DSM-5*-TR [2]. To evaluate insomnia severity, the severity levels are determined using the ISI questionnaire [45].

### Digital Study

This study is executed through a digital protocol designed to conduct a large-scale, longitudinal clinical trial with minimal staff resources. This protocol replaces the manual interventions of site-based studies with digital tools that manage the full participation process. The following sections detail each component, from initial recruitment to ongoing engagement and data management.

#### Recruitment

Participation in the study is contingent upon self-verification of several inclusion criteria. Participants must be individuals aged 18 years or older, be eligible for the French social security system, and must not be classified as vulnerable according to the French regulations in force [48]. The collection of informed consent is mandatory. Ownership of at least one Withings sleep-monitoring device compatible with the study, SW, SW2, or WSA, is also required, as is the installation of the WMA (version 5.12 or a more recent version) for the delivery of study questionnaires.

Individuals are excluded if they fail to meet any of these inclusion requirements. Further exclusion criteria, assessed through self-reported data, address potential threats to data integrity. These include sharing the device with another person, device use below a specified minimum frequency, or the presence of any known device malfunctions as detailed in Multimedia Appendix 1.

#### Engagement and Adherence Strategy

Sustaining participation in long-term decentralized trials presents a 2-fold challenge: ensuring not only high participant adherence (the consistent use of devices and the completion of surveys) but also deep engagement (the commitment to providing high-quality data). While traditional studies foster this commitment through personal contact, digital trials must use different strategies [49,50]. Our study uses a 3-pronged digital strategy to address both challenges. To cultivate the engagement necessary for thoughtful survey responses and proper device use, we educate participants on the importance of data integrity and apply gamification techniques. In addition, adherence is directly rewarded through financial incentives.

The initial participant onboarding process through the WMA is designed to mitigate the occurrence of low-quality data. To this end, the communications first establish the importance of valid research outcomes before detailing the 2 central requirements for participants: correct and regular device usage and the timely and accurate completion of questionnaires.

Additionally, scheduled nudges concerning the participants’ tasks (such as questionnaire completion, proper device usage, and device synchronization) are disseminated. The channels and timing of the nudges have been designed to address the trade-off between securing timely, high-quality data and minimizing disruption to participants’ daily lives. The schedule is shown in Figure 2. When a task is detected as incomplete, a sequenced notification strategy is initiated, beginning with subtle in-app reminders and escalating to more direct channels such as push notifications or emails if the task remains incomplete. More precisely, this sequence is triggered only after a set delay period of δ_1_ days following the task’s original due date and commences with an in-app notification within the WMA. If the task remains outstanding after a predetermined fixed temporal interval of δ_2_ days, a mobile device push notification is subsequently issued. Should the task still be incomplete after δ_3_ days, an email communication is sent.

**Figure 2.**
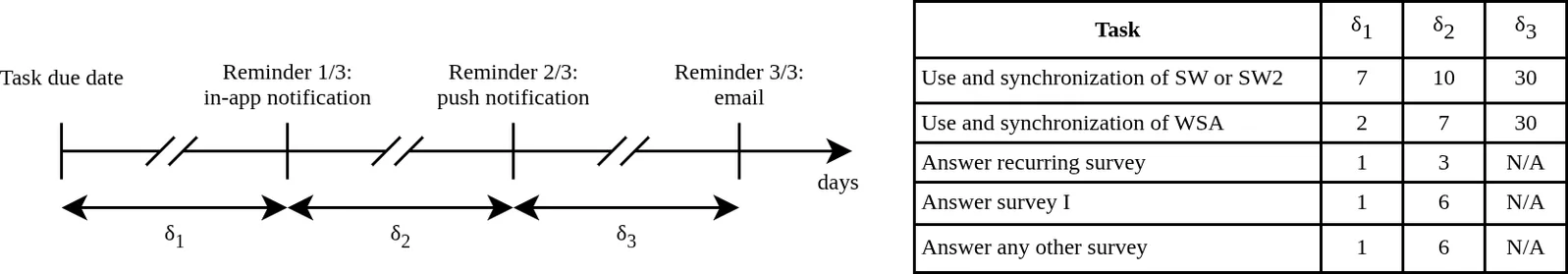
Schedule for the sequence of nudges for incomplete participant tasks. All parameters presented in the accompanying table for this figure are expressed in days. Not applicable (N/A) indicates that no such reminder is scheduled for the specified task. δ is the delay before scheduled nudges when a task has not been completed by the participant. SW: ScanWatch; SW2: ScanWatch 2; WSA: Withings Sleep Analyzer.

As gamification demonstrated to enhance adherence in health studies [51], the notification content integrated the participant progression, using the following metrics: the percentage completion of the individual’s participation duration, the cumulative number of questionnaire invitations sent to the participant, and the achievement of specific temporal milestones, such as reaching 50% or 75% completion of their total study involvement.

Since previous studies encourage the concomitant use of gamification and financial incentives to foster overall adherence [51], compensation has been incorporated. Eligibility for this financial benefit requires participants to meet 2 conjunctive criteria assessed over the participant’s entire 26-week involvement: first, at least one of the designated study devices must be used and successfully upload data on more than 60% of the nights (and 60% of the days for the watches), and second, more than 60% of all requested questionnaires must be completed. Eligible participants will be offered a choice between 2 forms of compensation: a €100 (€1=US $1.12 as of October 15, 2026) Withings discount coupon or a 6-month subscription to the Withings+service. The protocol uses a simple, all-or-nothing reward model: participants receive the full compensation if they meet predefined data submission thresholds and nothing otherwise. This structure was deliberately chosen over more complex systems because it offers 2 advantages: it is simpler to implement for the research team, and its rules are unambiguous for participants [52].

#### Withdrawal

To alleviate potential concerns regarding the long-lasting participation period and its substantial data collection demands, participants are informed of their unconditional right to withdraw consent at any time. The straightforward withdrawal process is detailed in the information notice and is consistently reiterated as a reminder at the end of each questionnaire.

To withdraw, participants contact Withings customer support via the designated email address or online help center, promptly receiving acknowledgment of their request’s receipt and initiation of processing. The maximum withdrawal processing period of 7 business days is outlined in the information notice and reiterated upon request.

For effective execution that is compliant with participant wishes and GDPR, participant withdrawals trigger a coordinated process. An automated participant identification system is used to prioritize communications from study participants over general customer inquiries. Customer support immediately relays requests to the data protection officer and the clinical study manager. The data protection officer ensures timely, compliant handling of the request, while the clinical study manager instructs the data management team to freeze or delete data per the participant’s request and to halt all study communications in the customer relationship management (CRM) database. The clinical study manager also provides final oversight, confirming all tasks are completed for formal closure.

#### Data Management

Given the scale and complexity of such a digital study, a robust data management plan is fundamental to yielding a reliable dataset. This plan is designed to ensure the collection of high-quality and reliable data while also upholding standards for data integrity, security, privacy, and availability across the entire data life cycle, from acquisition to final dataset preparation. This section outlines the automated procedures established to proactively address potential sources of data degradation or loss.

##### Data Security and Regulatory Compliance

To ensure rigorous data protection in this study, all data undergo pseudonymization and adhere to data minimization principles. Data storage occurs within secure, ISO 27001–certified environments, using robust encryption and strict access controls, with all activities logged and monitored. Regular security audits and risk assessments are conducted to verify ongoing compliance. All data handling practices fully comply with the GDPR and applicable French data protection laws. Furthermore, Withings maintains ISO 27701 certification for its Privacy Information Management System.

##### Data Acquisition and Quality Assurance

Following informed consent, specific device functionalities identified as insightful for sleep assessment are remotely activated: these include, for the WSA, features enabling the detection of snoring and sleep apnea and for the SW and SW2, the measurement of nocturnal peripheral oxygen saturation (SpO2). However, recognizing that these advanced features can cause inconvenience, such as increased battery consumption or concerns regarding microphone activation, the study prioritizes long-term participant retention over obtaining the richest possible dataset from every individual. Participants are therefore explicitly informed of their right to disable these specific functions, a choice that provides a less granular dataset but ensures their continued participation with core data collection intact.

The management of device firmware versions affects data quality throughout the study. This involves continuously ensuring firmware compatibility and consistency for all participants over the entire study duration. One concern is to mitigate potential risks to data uniformity that may arise from subsequent mandatory device updates, as these updates could alter the outputs of embedded data processing algorithms, thereby affecting the collected objective data. Instead of forcing participants onto a single, static firmware version that would compromise user security and experience, our primary mitigation strategy is to record the firmware version associated with every data point. This logged version information is essential for careful subsequent analysis, allowing for stratification or adjustment based on any firmware variations. This approach is supplemented by strategically timing the launch of new study cohorts relative to anticipated software update schedules.

To mitigate risks to data quality, a screening questionnaire is administered before final enrollment. This questionnaire is designed to exclude individuals when there is a high likelihood that they will generate unreliable data, a risk identified from two potential sources: (1) problematic anticipated usage patterns, such as device sharing or Bluetooth misconfiguration, and (2) device malfunctions identified using criteria derived from historical customer support data.

Because the recurring biweekly questionnaires assess the preceding 2-week period (Figures 1 and 2), prompt submission is essential to minimize recall bias. Therefore, participants are given a strict 6-day response window to ensure their answers accurately reflect this recent time frame. These windows also establish temporal consistency by standardizing data collection at regular, predefined intervals, which is required for longitudinal analysis. This structured approach also minimizes participant confusion by preventing multiple, potentially conflicting versions of a questionnaire within a brief time frame. Questionnaires not completed within this 6-day time frame are recorded as missed. This system also supports the criteria for equitably rewarding timely and consistent data provision.

To address potential data loss from device connectivity interruptions (eg, Bluetooth between SW/SW2 devices and smartphones, or Wi-Fi for WSA), which may still occur despite the participant-screening measures previously described, an automated monitoring system detects extended periods of absent data transmission. Upon such detection, scheduled reminders are dispatched to participants. The frequency of these reminders is calibrated based on each device’s internal storage capacity, aiming to prompt data synchronization before older, unsynced data is potentially overwritten by more recent recordings. These notifications also serve as general device-usage reminders when recent activity is lacking.

To ensure critical data transmission integrity within this study’s decentralized architecture, the reliability of the automated data upload system is continuously verified using dedicated monitoring tools. Transmission quality metrics have been established and tracked, including the volume of consecutive data points received, total data volume per participant, and the frequency and magnitude of data loss incidents.

##### Data Organization and Dataset Preparation

Previous studies [49] highlight the challenges associated with managing diverse data sources, such as questionnaire responses, device data, and withdrawal information. These challenges were addressed by implementing robust automated procedures. A primary function of these procedures is to ensure that all collected data are accurately linked to a unique, pseudonymized participant identifier. These procedures also establish unambiguous temporal ordering for every data point, irrespective of its source.

To ensure timely data availability, minimize data loss risk, and facilitate ongoing quality control, the study dataset is constructed progressively throughout the research period. This iterative method necessitates detailed upfront planning, including a detailed inventory of all data variables, their sources, retrieval mechanisms, and the defined dataset architecture. Every 3 days, an automated pipeline fetches new data from the user database, structures it according to this predefined architecture, and commits the output to a dedicated repository, managed by the open-source Git Large File Storage (LFS) extension [53], which serves as the study database. This process ensures version control and secure, incremental data aggregation.

Multimedia Appendix 2 states the prospective analysis plan for the participant-level classification of subjective insomnia presence, together with the anticipated forms of data incompleteness.

### Study Monitoring

Ongoing study monitoring sustains research integrity, data quality, and participant engagement and supports early pipeline and dashboard debugging. A primary aspect of this process is the active management of recruitment bias. While the study’s sampling population (Withings users) is distinct from the general population, thus creating a known selection bias, an objective of our monitoring is to minimize additional participation bias introduced by the recruitment protocol itself. To this end, we continuously benchmark the enrolled cohort’s characteristics, including demographic, clinical, and device-usage data, against the broader user database to detect imbalances, to enable timely investigation of protocol-related barriers, and to adjust recruitment strategies, ensuring the sample is representative of its source. This focus on the cohort’s internal validity will be complemented by a final characterization of its composition and biases (eg, insomnia prevalence), a prerequisite for valid subgroup comparisons and for training and evaluating ML models. This separate and broader challenge of generalizability to the general population will be addressed during the analysis phase. Multimedia Appendix 2 describes available adjustment methods.

Monitoring also guides other proactive and corrective measures, such as targeted recruitment of underrepresented subgroups, addressing high rates of participants disabling some device functionalities, and proactively managing participant adherence through trend analysis and revision of motivational content. Daily data updates are first structured by a model running on the open-source Data Book Tool (dbt Labs [54]). The resulting structured data is then fed into automatically updated dashboards on Metabase [55], another open-source platform. This data pipeline facilitates both data aggregation and visualization to enable data tracking every other day. This monitoring covers metrics systematically tracked across distinct domains of study execution and participant engagement.

Recruitment monitoring assesses the number of enrollments against predefined estimates (as detailed in the *Sample Size Estimation: Determining Necessary and Achievable Cohort Size* section) shown in Figure 3, the estimated number of the remaining potential participants, the proportion of participants who have been excluded by the study’s screening criteria, and the yield from different recruitment communication channels. These criteria, developed from user issues reported via customer support, are specifically monitored to ensure they are not unduly restrictive. Participant cohort characteristics are monitored to identify and address potential cohort imbalances, with ongoing monitoring assessing the distribution of enrolled participants across demographic and clinical profiles, as well as 2 nonmutually exclusive device-usage groups—specifically, individuals using at least one smartwatch (SW or SW2) and those using at least one WSA. These distributions are then benchmarked against a predefined target population profile derived from the Withings CRM database. Illustrations are shown in Figure 4 and in Figure B1 of Multimedia Appendix 2. Questionnaire engagement is tracked through completion rates and intraquestionnaire drop-off points (see the example in Figure 5). Adherence to device functionalities is tracked through the rates at which participants maintain active use of or choose to disable the device functionalities automatically enabled at consent, as explained in the *Data Acquisition and Quality Assurance* section. Compensation eligibility is tracked through the number of participants achieving it.

**Figure 3.**
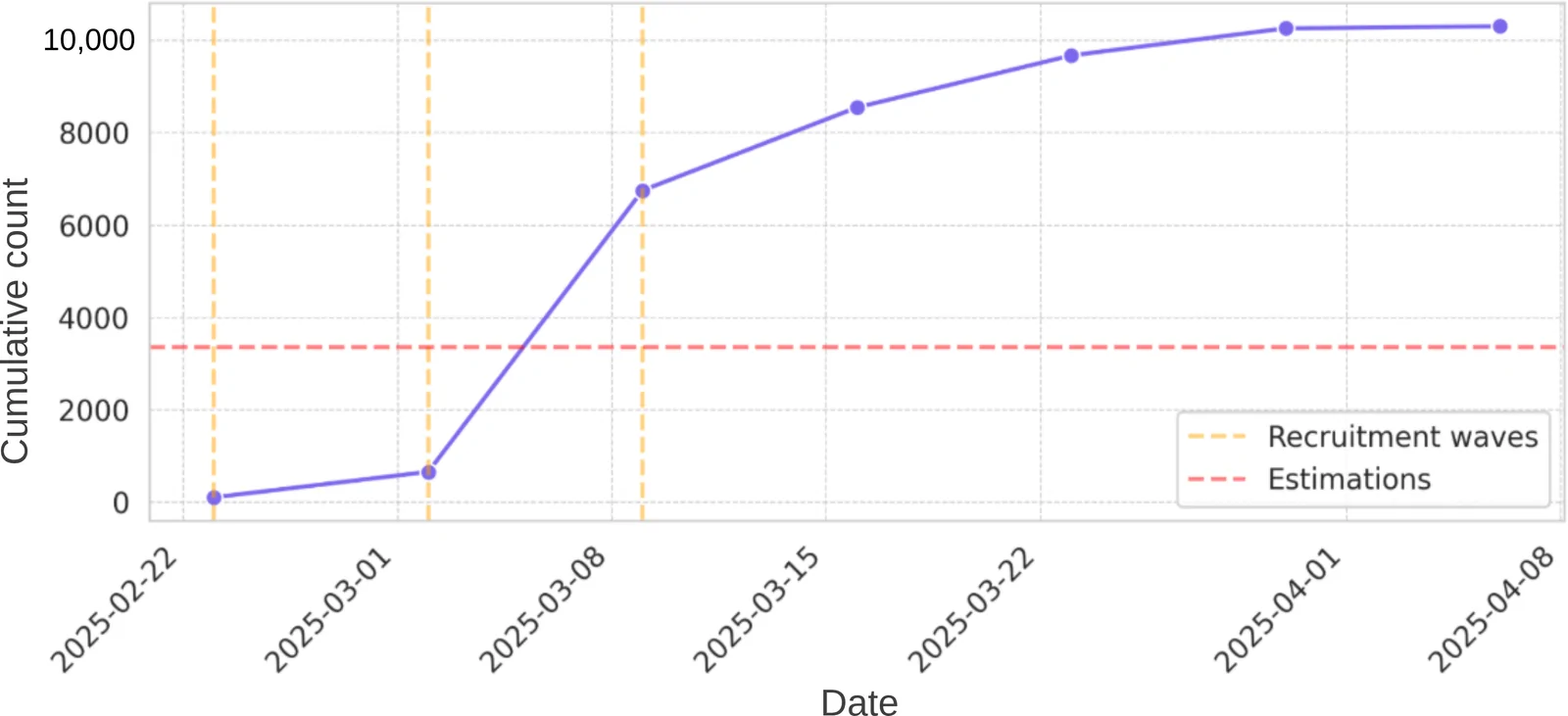
Comparison of actual and projected participant inclusions. Cumulative participant enrollment (solid blue line) is shown against the initial projection of approximately 3400 (dashed red line). The increases correspond to 3 recruitment waves targeting cohorts of approximately 2000, 10,000, and 50,000 users, as detailed in the *Methodological Validation and Phased Implementation* section.

**Figure 4.**
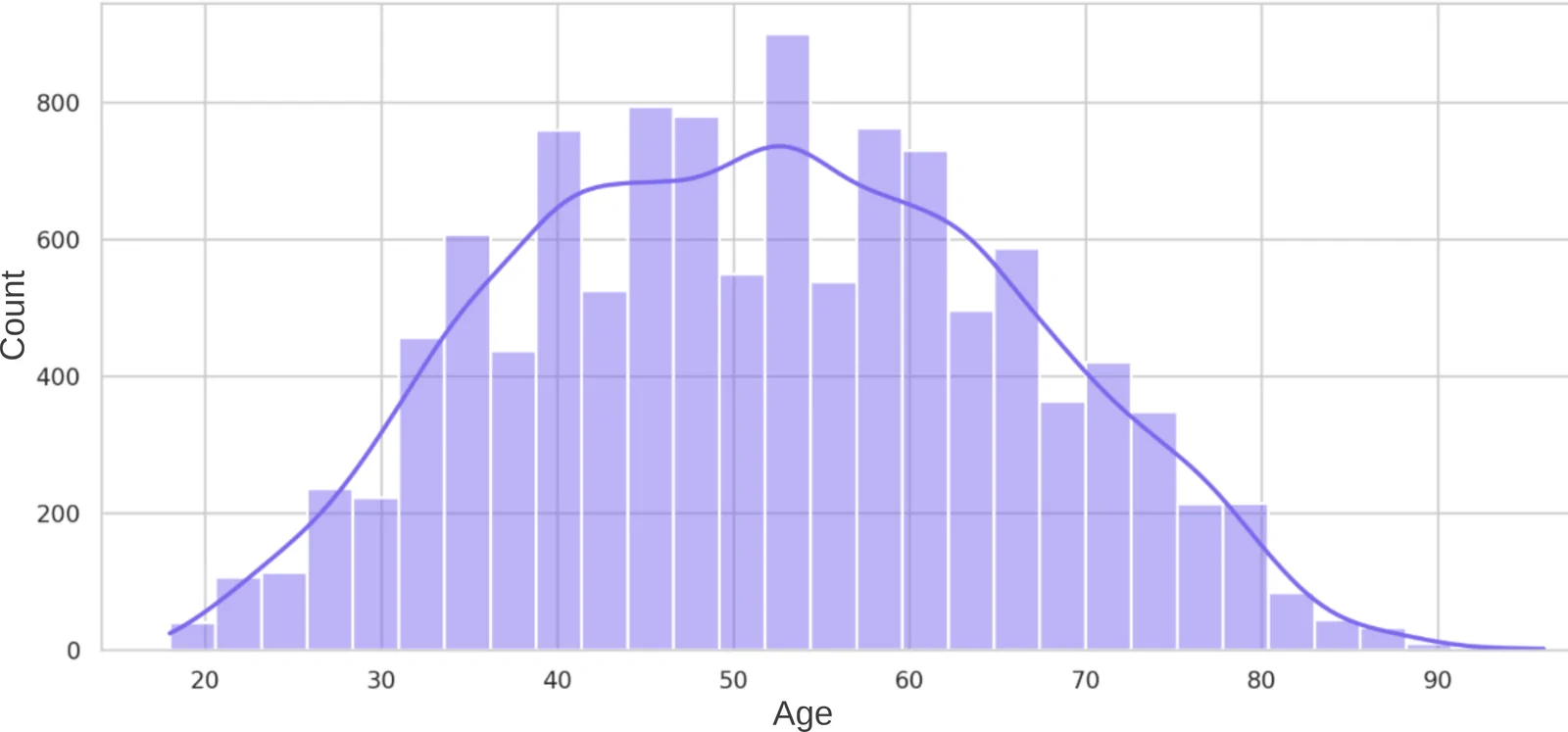
Distribution of participants’ ages. The blue line is the kernel density estimate.

**Figure 5.**
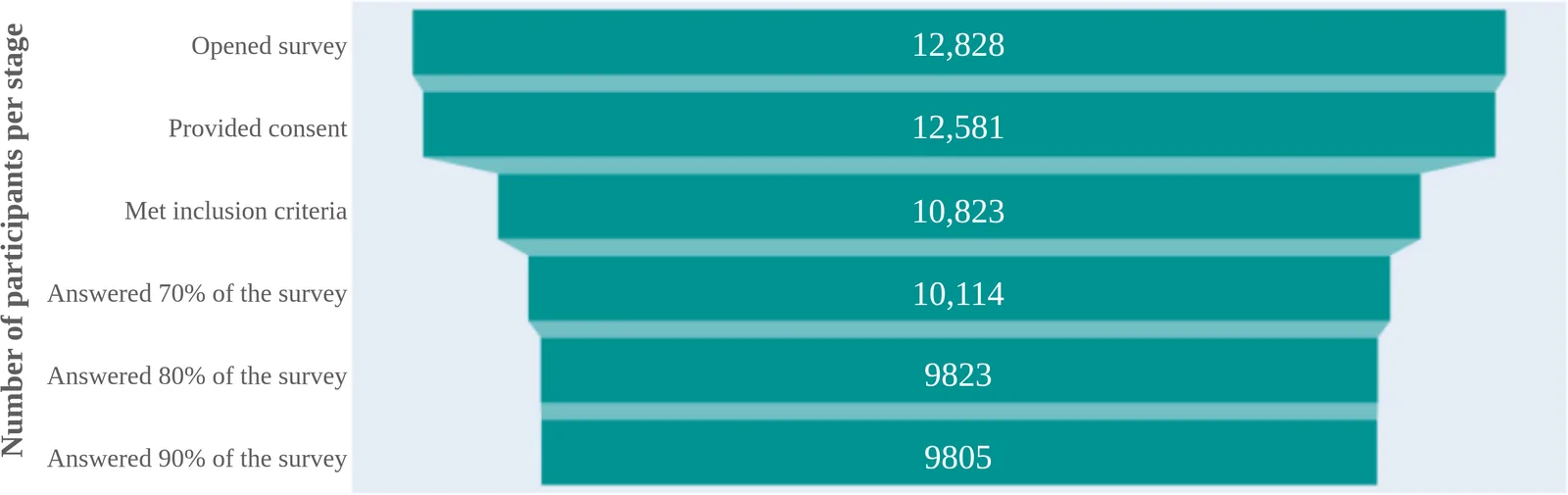
SLEEPI initial survey: participant completion funnel. Illustration of participants’ drop-off points. A total of 62,000 users were contacted.

### Sample Size Estimation: Determining the Necessary and Achievable Cohort Size

The primary aim of this exploratory study is to generate a large-scale, longitudinal dataset of sufficient diversity to train ML models for insomnia assessment from the ground up. Because the objective is the discovery of generalizable patterns rather than the testing of a single, prespecified hypothesis, conventional sample size calculations are methodologically inappropriate for this data-driven research paradigm. To estimate the dataset size required to train a model, we conducted an a priori power analysis using Monte Carlo simulations, whose data-generating process, model specifications, and success criteria are detailed in Multimedia Appendix 2. The analysis determined, for several performance targets, the sample size at which statistical power reaches at least 80%, as shown in Figure 6. Based on these results, we concluded that about 1000 participants are sufficient to train a standard ML model such as a support vector machine and achieve satisfactory performance for this task.

**Figure 6.**
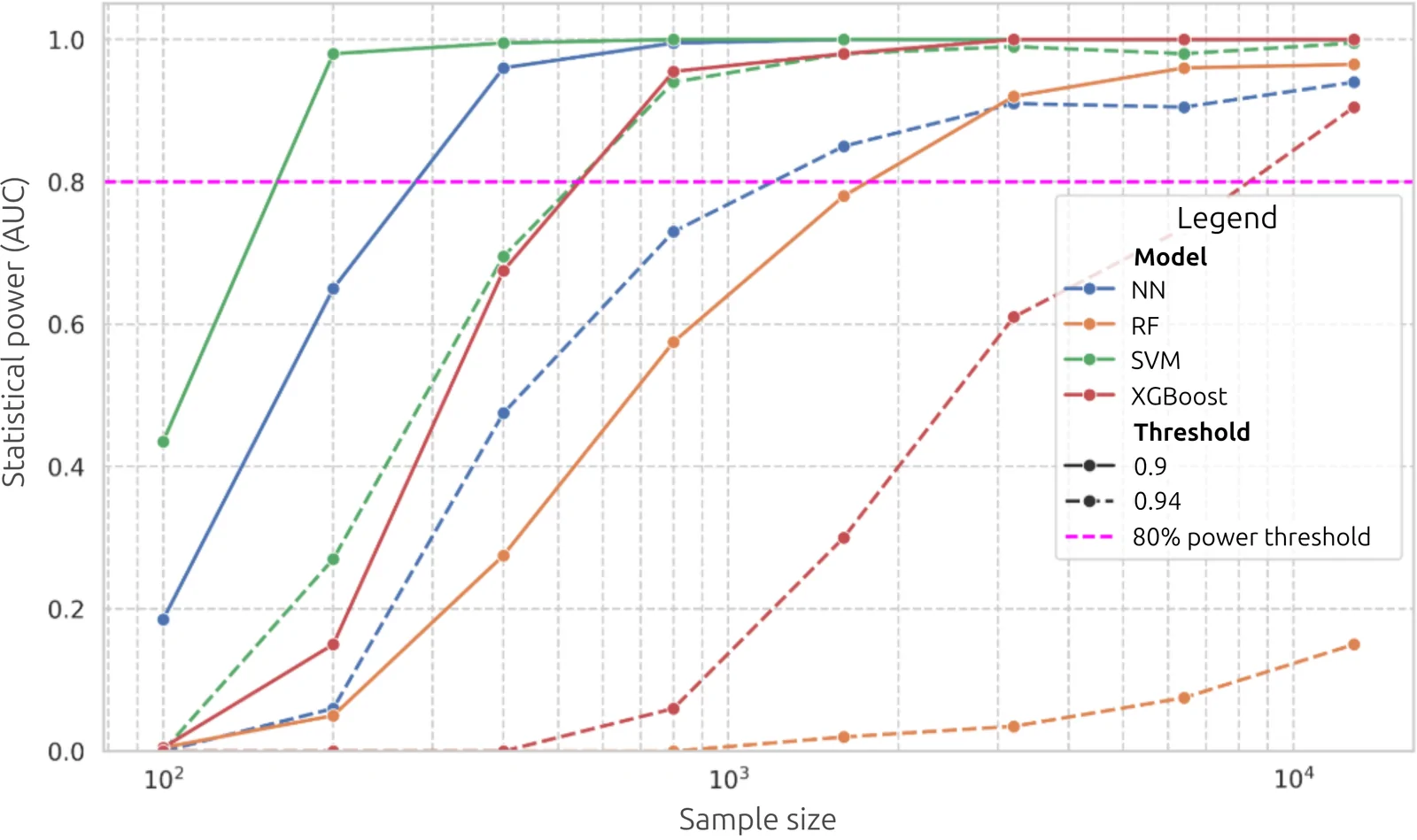
Power curves obtained through simulation for subjective insomnia classification using different area under the curve (AUC) success criteria. NN: neural network; RF: random forest; SVM: support vector machine; XGBoost: extreme gradient boosting.

Having established a target for the necessary sample size, we developed a prospective attrition model to determine whether this target is achievable and to calculate the required number of initial contacts. Its stages, retention rates, and sequential calculation are detailed in Multimedia Appendix 1. The model projects that about 83,662 users must be contacted to include 3175 participants and eventually yield 1000 usable datasets for the analyses, as illustrated in Figure 7.

**Figure 7.**
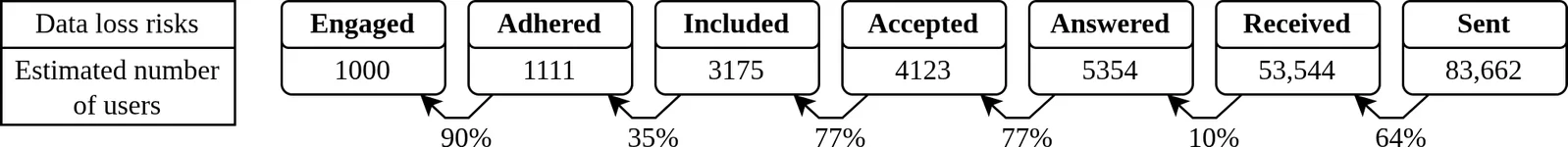
Illustration of the prospective attrition model.

The figure of 1000 participants represents the minimum usable analysis set, not the enrollment target. The simulation assumed complete data on every participant, whereas the collected data are sparse and subject to attrition across the 26-week monitoring period, so enrollment is sized above that floor to preserve both the size and the demographic diversity of the analysis set. Planned recruitment comprises the initial wave, closed with 10,823 participants, and a single deferred SW2 wave detailed in the *Methodological Validation and Phased Implementation* section. Roughly a quarter of the 25,000 SW2 users to be contacted are expected to consent, an approximation resting on the fact that this device is the most recently commercialized of those included in the study; thus that wave is expected to add about 6000 participants for a planned total of approximately 17,000. The protocol was amended accordingly. No further wave is planned. Recruitment ends with the close of the inclusion window, which is capped at 26 weeks in total; the initial wave used 6 of them, leaving 20 weeks.

### Methodological Validation and Phased Implementation

To ensure the robustness of the study procedures prior to and during full-scale operation, a sequence of validation steps and a phased implementation approach were adopted. The different phases are summarized in Table 1.

**Table 1.** Summary of study validation and phased implementation.

Phase name	Sampling population	Number of targeted participants	Time to respond	Objectives
Technical validation	Withings employees	18	1 month	Identify and resolve bugs in automated systems (questionnaires and data pipeline) Validate device feature activation Finalize study monitoring dashboards
Protocol refinement	Withings employees	80	1 month	Assess and refine the clarity of all participant-facing materials Generate preliminary attrition estimates Evaluate initial engagement, adherence, and questionnaire burden
Initial study rollout and operational validation (part 1/2)	Withings users	2000	1 week	Verify the accuracy of CRM^a^-based participant targeting at scale Assess system performance, stability, and scalability under real-world load Refine the withdrawal process based on user feedback
Initial study rollout and operational validation (part 2/2)	Withings users	10,000	1 week	Verify the accuracy of CRM-based participant targeting at scale Assess system performance, stability, and scalability under real-world load Refine the withdrawal process based on user feedback
Main study launch	Withings users	50,000	6 weeks	Execute the main phase of long-term data collection with the initial device cohorts (SW^b^ and WSA^c^)
Final study launch	Withings users	25,000	N/A^d^	Execute the main phase of long-term data collection with remaining device cohort (SW2^e^)

a CRM: customer relationship management.

b SW: ScanWatch.

c WSA: Withings Sleep Analyzer.

d N/A: not applicable.

e SW2: ScanWatch 2.

#### Testing Phases

The value of pilot testing in large-scale digital health research, particularly for validating data systems including data flow, integration, and integrity, is well-established [49]. Informed by such findings and to prepare for the main trial, 2 distinct phases of testing and protocol refinement were conducted exclusively among Withings employees.

The first phase of technical validation included 18 participants over 1 month. Its objectives were to identify and resolve significant bugs within automated study processes. Testing encompassed the questionnaire and reminder deployment system, detailing its scheduling and conditional logic; the data management pipeline, specifying its functions for data acquisition, integrity checks, linkage, and initial dataset–building routines; and device feature activation protocols. This initial phase also finalized the development and initial validation of the study monitoring dashboards.

Subsequently, a larger refinement phase was conducted with 80 participants, also for 1 month. An essential characteristic of this cohort was that the majority of individuals had no prior knowledge of the study’s existence, goals, or design, making them ideally suited for this phase’s primary focus: assessing and refining the clarity of all participant-facing materials. Particular attention was given to ensuring that study criteria, device usage guidelines, and compensation information were unambiguous for individuals new to the study’s specific requirements. Further objectives included generating preliminary attrition estimates, evaluating initial engagement with the long baseline questionnaire, and assessing early adherence patterns and the associated questionnaire burden arising from the first recurring questionnaires.

#### Initial Study Rollout and Operational Validation

Following the testing phases, large-scale recruitment was initiated, rolling out to 12,000 targeted individuals in 2 successive waves: an initial 2000 followed by 10,000. While this phase marked the formal start of data collection for the primary study objectives, it concurrently served as a period of real-world operational validation and refinement at scale.

The objectives during this initial scaled implementation were to verify the accuracy of participant targeting achieved through the internal CRM tool’s population segmentation; assess the performance, stability, and scalability of all study systems under increased load, including recruitment pipelines, participant engagement throughout the initial long questionnaire, and adherence to recurring questionnaire schedules; further refine the participant withdrawal process based on real-world usage and feedback; and identify and promptly resolve any residual technical issues or bugs specific to the production environment before extending recruitment to the remainder of the target population.

The study’s recruitment is deliberately structured in distinct phases to manage firmware updates across devices. For instance, a group of 25,000 SW2 users is reached out to in a later recruitment wave because of the need to release new device firmware and to give enough time for the users to make the update.

The study procedures were fixed in the protocol approved before enrollment opened. The 2 testing phases were operational tests conducted outside the trial. The refinements made during the initial rollout concerned automated processes and data storage; they altered neither the information notice nor the consent materials, and had no effect on participants. Changes affecting the protocol were submitted as amendments and approved by the Ethics Committee, and none were applied before that approval.

## Results

The SLEEPI study was initiated on February 18, 2025. During the initial phase of participant recruitment, 62,000 Withings users were contacted, resulting in 10,823 unique individuals included within the first 6 weeks. This represents a contact-to-inclusion conversion rate of approximately 17%, with only 4 participants having withdrawn. No further inclusions occurred after this 6-week period. A later recruitment wave of 25,000 SW2 users is scheduled to open during winter 2026 to 2027, and data collection is projected to end in June 2027. No deviations from the approved protocol have occurred.

Recruitment proceeded in 3 successive waves, contacting 2000 users, then 10,000, then 50,000, which together made up the 62,000 users contacted during the initial phase (Table 1).

These inclusion figures surpassed the study’s original projections. This initial projection, formulated at the time of protocol design, anticipated a total of 3354 inclusions over a 26-week period. Individuals who owned multiple devices were counted more than once. The projection was based on an estimated total pool of approximately 84,000 contacted users, which included the SW2 cohort, who were excluded from the initial recruitment wave. For the detailed calculations about the projections, please refer to Multimedia Appendix 1.

This higher-than-projected enrollment (Figure 3) stemmed from 3 stages where the initial attrition model proved overly conservative. The response rate to the initial outreach was higher than anticipated (16% vs a projected 10%), likely driven by user interest in sleep research and the provided financial incentive. Among those who responded, the rate of consent to participate was greater than expected (96% vs a projected 77%). Following consent, the rate of individuals meeting the final inclusion criteria was also higher than expected (90% vs a projected 81%).

Recruitment during the initial phase surpassed its projections at all 3 stages and prompted a temporary pause to revise the study budget. The pause was a budgetary decision, and the recruitment and data collection systems absorbed the observed inclusions over 6 weeks without operational failure. Preliminary analyses of the first recruitment wave have been conducted. Analysis of the complete dataset will follow the end of data collection. The first results are expected to be published in the summer of 2027, and the analysis of the entire dataset in the summer of 2028.

## Discussion

### Clinical Implications

The implementation of the SLEEPI study at scale provides a foundational step toward integrating these digital health technologies into clinical practice. Such integration could enhance multiple facets of patient care, from enabling large-scale screening and improving diagnostic accuracy to facilitating longitudinal monitoring and refining patient phenotyping for more personalized treatment. The continuous collection of objective data allows for the assessment of intraindividual variability in physiological and behavioral metrics, while the concomitant collection of subjective data offers insights into symptom fluctuations [12].

### Limitations of the Study Design

#### Challenges of Datasets Derived From Consumer Devices

The use of consumer-grade devices in free-living conditions is a deliberate methodological choice that prioritizes scalability, user experience, cost-effectiveness, and longitudinal data collection over the precision attained in a clinically controlled environment. To enable passive, automatic, long-term collection of data, consumer-grade smart devices sometimes use proxies (eg, pressure variations in a bladder caused by chest movements in place of impedance belts) and fewer sensors than some gold-standard techniques such as polysomnography. Furthermore, variable participant adherence to device-use instructions leads to more noise and missing values in decentralized trials than in centralized studies. Consequently, occurrences of data loss or intermittent gaps are anticipated.

Furthermore, the study cohort, composed primarily of existing Withings device users, may not fully mirror the broader general population. This sampling approach can introduce selection biases; for example, the cohort might differ in terms of socioeconomic status, technological proficiency, or baseline health awareness. Such biases could limit the generalizability of observed patterns and outcomes to more diverse demographic groups. At the analysis stage, as detailed in Multimedia Appendix 2, generalizability to the general population will be assessed and, where appropriate, the cohort reweighted to published population totals by poststratification or raking, with model performance reported both with and without this adjustment; because such reweighting accounts only for measured characteristics, residual bias from unmeasured differences remains possible.

A further constraint concerns the reference labels. They are collected without a clinical interview; for example, a comorbid sleep or mental disorder that has never been diagnosed can neither be identified nor ruled out as a better explanation of the complaint, and any discrepancy between a participant’s perception of their sleep and its physiological measurement is carried over into the label. Model performance will therefore be measured as agreement with participant-reported insomnia; it is not a measure of diagnostic accuracy against clinical evaluation.

#### Trial Digitalization: A Foundational, Resource-Intensive Infrastructure

Maintaining long-term participant adherence and engagement in this decentralized study presents significant challenges to personalized, proactive interventions, in contrast to more traditional site-based study designs that rely on direct contact with health care professionals for support and assessment. Consequently, the study relied on clear materials, precise instructions, and automated reminders to guide participants while strategically balancing information provision to avoid overwhelming them.

Implementing this decentralized study necessitated a substantial initial investment to develop and adapt existing Withings tools and resources into reusable foundational infrastructure that includes robust data management systems, adaptable study design protocols, and defined workflows covering recruitment, participant withdrawal, and phased implementation strategies. Although the initial setup was resource-intensive, these core developments are reusable across other health domains.

### Future Work

The methodology for decentralized trials could be advanced through subsequent research in several areas identified by this study. First, developing an in-app participant interface for real-time progress tracking could improve clarity and support more advanced gamification strategies. Second, systematic A/B testing is recommended to empirically optimize engagement approaches, such as CRM content and reminder protocols [56,57]. Third, the rich longitudinal dataset collected warrants in-depth analysis to characterize long-term patterns of participant adherence and engagement with both devices and questionnaires, thereby identifying predictors of sustained participation. Finally, future protocols could incorporate methods to characterize and statistically adjust for the selection biases affecting commercial user populations, thereby enhancing the generalizability of findings to the broader public.

## Conclusions

This study presents a protocol for a large-scale, decentralized trial using consumer smart devices to identify physiological signatures of subjective insomnia. This protocol addresses the increasing need for large, longitudinal datasets essential for advanced analytics, such as ML approaches, a requirement that is often challenging for traditional research paradigms and sample size methodologies.

Methodological contributions include a 2-part sample size strategy that first uses simulation to define the minimum sample size required for the ML objective and then uses a prospective attrition model, based on clinical trial forecasting [58], to calculate the number of initial contacts needed to achieve that target. Furthermore, the overall design for this large-scale, digital, longitudinal, and decentralized trial is described in sufficient detail to be replicated or adapted, covering recruitment from an existing user base, data quality assurance, participant engagement, and robust data management.

While decentralized studies of this scale face the challenges detailed in the *Limitations* section, this work reports the practical decisions and procedures used for study management and monitoring. While this trial benefited from an established digital infrastructure, its core methodologies are intended for adaptation by other organizations conducting large-scale real-world data studies. However, such adaptation would necessitate a careful assessment of the distinct resource requirements, particularly for participant recruitment, device provisioning, and ongoing support, especially for entities lacking a comparable preexisting infrastructure.

Ultimately, this methodological development is driven by the clinical need for more nuanced, objective tools to support specialists in insomnia diagnosis and care. By seeking physiological signatures grounded in data that can be correlated with clinically validated subjective assessments [2,45-47], the aim is to develop technologies that complement existing clinical pathways. Thus, this study contributes to insomnia research and sets out a methodological approach intended to translate real-world digital health data into clinically relevant insights, with potential applicability to other health domains.

## Supplementary material

10.2196/92341 Multimedia Appendix 1 Identification and assessment of data loss sources and prospective attrition model.

10.2196/92341 Multimedia Appendix 2 Methods for the a priori power analysis conducted to determine the necessary sample size for the study’s machine learning objectives.

## Abbreviations

CPP: Comités de Protection des Personnes
CRM: customer relationship management
DCT: decentralized clinical trial
*DSM-5*-TR: *Diagnostic and Statistical Manual of Mental Disorders*, Fifth Edition, Text Revision
GAD-7: Generalized Anxiety Disorder-7
GDPR: General Data Protection Regulation
ISI: Insomnia Severity Index
LFS: Large File Storage
ML: machine learning
PHQ-9: Patient Health Questionnaire-9
PRO: patient-reported outcome
SLEEPI: Smart Devices-Based Longitudinal Assessment of Patterns of Insomnia
SpO2: peripheral oxygen saturation
SW: ScanWatch
SW2: ScanWatch 2
WMA: Withings mobile app
WSA: Withings Sleep Analyzer
