# Supplementary material for: Smart Devices–Based Longitudinal Assessment of Patterns of Insomnia (SLEEPI): Protocol for Large-Scale, Digital, Longitudinal, and Decentralized Clinical Trials
Source: JMIR Res Protoc. 2026 Nov 2;15:e92341. doi: 10.2196/92341 (PMC13737349; doi:10.2196/92341)
Supplement: Supplementary file 1 [file resprot-v15-e92341-s002.docx]

Supplementary Materials B

This document details the methodology for the a priori power analysis conducted to determine the necessary sample size for the study's machine learning objectives. The process consists of three core stages: defining a Data Generating Process (DGP), optimizing model hyperparameters, and running the final Monte Carlo power analysis. Determining the necessary sample size to train an ML model is driven by the principles of statistical learning. A training dataset must be of sufficient scale and diversity, exposing the model to a wide variety of examples and configurations across all relevant subgroups, to capture the complexity of the problem, and therefore ensure it can develop robust and generalizable capabilities. To ensure an ML model can achieve high performance, we first need to estimate the required dataset size. To establish this estimate, we employed a composite approach grounded in theory, literature, and direct simulation.

While theory provides some guidance for simple classifiers [[1,2]](https://www.zotero.org/google-docs/?8uNogY), there is no universal rule for complex, non-linear models. The literature suggests that simpler models (e.g., SVM, Random Forests) often require about thousands of samples, while more complex architectures (e.g., XGBoost, Neural Networks) require tens of thousands [[3–6]](https://www.zotero.org/google-docs/?0beeQd). These figures are rules of thumb, as the exact number is highly contingent on the specific properties of the dataset and the chosen model. Dataset characteristics include the signal-to-noise ratio (SNR), overall dimensionality, the quantity and quality of features (including their correlation with the outcome), and the degree of class separability. This is further influenced by the specific parameters of the model architecture, which are themselves tuned based on the collected data.

To derive an estimate specific to our use case, we conducted an a priori power analysis using Monte Carlo simulations [[7,8]](https://www.zotero.org/google-docs/?90Xuoc). This standard approach involves a sequence of steps: first, we defined a data-generating process to create synthetic training and testing sets that mimic our expected data. Second, a success criterion was established, defined as the ability of a model to distinguish between insomnia and healthy subjects with a given Area Under the Curve (AUC) on the test set.

Further sections describe the forms of data incompleteness anticipated in the collected dataset, the prospective analysis plan, the selection bias affecting generalizability, the distribution of device ownership among enrolled participants, and the recruitment procedures.

# 1. Data Generating Process (DGP)

To perform the simulation, a Data Generating Process (DGP) was designed to create synthetic datasets based on a multivariate logistic regression framework [[9]](https://www.zotero.org/google-docs/?NGkI2U), mimicking the anticipated complexity of the real-world study data.

The process began by generating 50 latent continuous features from a multivariate normal distribution. This step was governed by a 50x50 correlation matrix with a realistic, randomized mix of weak (∣ρ∣≤0.1), moderate (0.1<∣ρ∣≤0.4), and strong (0.5≤∣ρ∣<1.0) inter-feature correlations to simulate multicollinearity. A subset of these latent variables was then transformed to create the final feature set, composed of 40 continuous and 10 four-level categorical variables.

The binary outcome was simulated to a 30% prevalence [[10,11]](https://www.zotero.org/google-docs/?Pl75Ur) using a logistic function. The predictive strength of the relationship between the features and the outcome was controlled by a set of 50 predefined coefficients, which were shuffled and randomly assigned to the features. This set was composed of three tiers of effect sizes: 5 strong (drawn from a uniform distribution in the range [1.2, 2.0]), 25 moderate (range [0.5, 0.8]), and 20 slight (range [0.01, 0.1]).

Finally, to test model generalizability to unseen subjects, each simulated sample was assigned to one of two distinct groups to allow a Leave-One-Group-Out cross-validation during hyperparameter tuning.

# 2. Model Specifications and Hyperparameter Tuning

Four machine learning models were selected for evaluation: a Support Vector Machine (SVM), a Random Forest (RF), an XGBoost model, and a Neural Network (NN).

As a preliminary step, a hyperparameter tuning process was conducted for each model architecture to identify the optimal set of parameters for this specific data structure. This tuning was performed using *GridSearchCV* on a dedicated synthetic dataset of 500 samples. The grids of parameters explored and the resulting optimal set of hyperparameters for each model are detailed in Table B1.

| **Model** | **Parameter** | **Values Explored** | **Optimal Value** |
| --- | --- | --- | --- |
| **SVM** | kernel | rbf, linear | rbf |
|  | C | 1, 10, 100 | 100 |
|  | gamma | 0.1, 0.01, scale | 0.01 |
| **Random Forest** | n_estimators | 100, 200 | 100 |
|  | max_depth | 10, 20, None | 10 |
|  | min_samples_split | 2, 5 | 2 |
|  | min_samples_leaf | 1, 2 | 2 |
|  | max_features | sqrt, 0.5 | sqrt |
|  | bootstrap | TRUE | TRUE |
| **XGBoost** | n_estimators | 100, 200 | 200 |
|  | learning_rate | 0.05, 0.1 | 0.1 |
|  | max_depth | 3, 5, 7 | 3 |
|  | subsample | 0.8, 1.0 | 0.8 |
|  | colsample_bytree | 0.8, 1.0 | 1 |
|  | gamma | 0, 0.1 | 0 |
|  | reg_alpha | 0, 1 | 0 |
|  | reg_lambda | 1, 10 | 1 |
| **Neural Network** | hidden_layer_sizes | [50], [100], [50, 30] | [100] |
|  | activation | relu | relu |
|  | solver | adam | adam |
|  | alpha | 0.0001, 0.001 | 0.0001 |
|  | learning_rate_init | 0.001 | 0.001 |
|  | early_stopping | TRUE | TRUE |
|  | validation_fraction | 0.1 | 0.1 |

Table B1: Model Architectures, Hyperparameter Tuning Grids, and Optimal Values

# 3. Monte Carlo Power Analysis

Using the optimal hyperparameters identified above, the main power analysis was conducted via Monte Carlo simulation. The simulation was executed for a series of eight distinct sample sizes — 100, 200, 400, 800, 1,600, 3,200, 6,400, and 12,800 — with a total of 200 simulation loops performed for each.

In each loop, a new dataset was generated via the DGP, and a model was trained using its pre-defined optimal parameters. To assess generalization, the model's performance was evaluated on a dedicated, held-out test set of 10,000 samples, which was independently generated within each simulation loop. For a given sample size, statistical power was calculated as the proportion of the 200 simulation runs where the model met a predefined success criterion, such as achieving an Area Under the Curve (AUC) of at least 0.90, 0.92, or 0.94.

The AUC is a measure of the model’s discrimination. For clinical interpretability, we also calculated the sensitivity and specificity for each model. This requires applying a classification threshold (e.g., 0.5) to the model's continuous probability score to convert it into a binary prediction (insomnia or healthy). The mean accuracy, sensitivity, and specificity values for each model are detailed in Table B2.

| **Model** | **Minimum Sample Size** | **Mean accuracy* (std)** | **Mean sensitivity* (std)** | **Mean specificity* (std)** |
| --- | --- | --- | --- | --- |
| **SVM** | 800 | 0.88 (0.01) | 0.85 (0.03) | 0.90 (0.02) |
| **NN** | 1600 | 0.88 (0.01) | 0.86 (0.03) | 0.89 (0.02) |
| **RF** | 3200 | 0.83 (0.02) | 0.75 (0.04) | 0.90 (0.01) |
| **XGBoost** | 800 | 0.84 (0.01) | 0.80 (0.03) | 0.87 (0.02) |

Table B2: Simulated Minimum Sample Size and Performance Metrics for Insomnia Classification

* Computed as the mean of each metric obtained on all simulations

The simulated performance metrics for our models, with accuracy ranging from 0.83 to 0.88, sensitivity from 0.75 to 0.86, and specificity from 0.87 to 0.90, are comparable to the range of those reported in the existing literature [[12–16]](https://www.zotero.org/google-docs/?bU7eC9) (accuracy: [0.73, 0.98]; sensitivity: [0.76, 0.92]; specificity: [0.82, 0.91]).

The simulation results for sample efficiency were unexpected: while we anticipated a performance ranking of Random Forest, then XGBoost, then Neural Network (NN), our findings showed that XGBoost and the NN required less data than the Random Forest. This outcome can be explained by the interaction between the models' core architectures and the specifics of our experimental design.

The NN's high performance at small sample sizes is likely due to its architectural suitability for the problem. Because the synthetic data was generated using a logistic regression model, the true underlying relationship between features and the outcome is smooth. The NN, being a universal function approximator, is good at learning such smooth decision boundaries. In contrast, tree-based models like XGBoost and Random Forest create step-wise, "boxy" boundaries, which are less efficient at capturing this specific type of relationship.

Regarding tree-based models, RF model’s low performance and XGBoost's high performance with small sample sizes can be attributed to the hyperparameter tuning being performed on a small, fixed sample size of 500. This process locked XGBoost into a "weak learner" configuration (shallow trees), which reduces bias on small datasets. Conversely, the Random Forest was locked into a "strong learner" configuration (deeper, high-variance trees) that is prone to overfitting and requires a much larger dataset to effectively reduce variance through its bagging process.

# 4. Anticipated Data Incompleteness

Two forms of incompleteness are anticipated in the collected dataset. The first is the absence of a variable for the whole study duration, which occurs when a participant exercises the right to disable a functionality activated at consent, namely nocturnal SpO2 measurement on the ScanWatch and ScanWatch 2 or snoring and sleep apnea detection on the Withings Sleep Analyzer. The second is an intermittent gap within a variable that is otherwise collected, arising from nights or days without device use, from connectivity interruptions, and from unanswered questionnaires.

Both forms are quantified during data collection by the metrics of the Study Monitoring section of the main text: the rate at which participants disable functionalities, and the frequency and magnitude of data loss incidents among the transmission quality metrics.

Section 5 states how each form is handled in the primary analysis.

# 5. Prospective Analysis Plan

This section states the procedures and decision rules of the primary analysis. Any departure from this plan will be reported with the analysis, together with its reason.

The primary analysis is a binary classification of insomnia presence at the participant level, on the analysis set defined in Supplementary Materials A. The questionnaire implementing the DSM-5-TR criteria is administered at inclusion and every 3 months thereafter, giving three administrations across the 26-week monitoring period. A participant is labeled a case when the criteria are met at a majority of the administrations answered, and a participant answering two administrations that disagree is labeled a case. Participants answering fewer than two administrations are excluded from the primary analysis for want of a label. The comparison class contains every other participant, including those reporting a sleep disorder other than insomnia. The proportion of cases observed will be compared with the 30% prevalence assumed in section 1.

Two sensitivity analyses accompany it. The first restricts the evaluation to participants whose administrations agree. The second excludes participants for whom the devices detect moderate to severe sleep apnea.

The predictors are limited to variables derived from the device sensors and to demographic variables. Responses to the severity, depression and anxiety questionnaires are excluded from the predictors, so that performance reflects the sensor data rather than the agreement between questionnaires answered by the same participant. Each sensor variable is summarized over the monitoring period by a measure of central tendency and by the within-participant standard deviation across nights, and further variables may be derived from the activity and sleep-wake data. The number of predictors admitted to a model will be constrained by supervised feature selection applied within the training data alone, so that the dimensionality stays close to the 50 features assumed in section 1 and the sample-size analysis continues to apply [[2]](https://www.zotero.org/google-docs/?nDBRId).

Participants are partitioned into training, validation and test subsets in the proportions 70%, 15% and 15%, stratified on the label, on sex, on age group and on body mass index group, and the partition is established before any model is fitted. No participant appears in more than one subset. Every quantity estimated from data, including standardization constants, the values used to fill absent variables, the selection of features, the model hyperparameters and the classification threshold, is estimated within the training data alone. For the primary analysis the label and the predictors summarize the same monitoring period.

Section 4 describes two forms of incompleteness. A variable absent for the whole monitoring period, which occurs when a participant disables a functionality or does not own the device that measures it, is filled with a constant and accompanied by an indicator variable recording its absence, so that a model can use the absence itself where it carries information [[17]](https://www.zotero.org/google-docs/?zXzSCJ). An intermittent gap within a variable that is otherwise collected requires no imputation, because each summary is computed over the nights on which the variable was recorded.

No resampling and no class weighting are planned. Cases are expected to represent 30% of participants, which is not a severe imbalance.

Five model classes are compared: a penalized logistic regression, which serves as the baseline a more flexible model must outperform to be preferred, together with the support vector machine, random forest, XGBoost and neural network specified in section 2. Hyperparameters are selected by a search over ranges stated before the analysis begins, conducted by cross-validation within the training data, with all data from one participant kept within a single fold. The model retained is the one reaching the highest mean AUC across those folds.

Accuracy, sensitivity and specificity are defined only once a predicted probability is converted into a binary decision. The threshold is set on the validation data at the value maximizing the sum of sensitivity and specificity, which is the Youden index. No level of performance is set as a target, the study being exploratory.

The primary metrics are accuracy, sensitivity and specificity, reported with the confusion matrix and with 95% confidence intervals on the test data. The AUC is reported as a complementary measure of discrimination. The agreement between the predicted probabilities and the observed frequencies is reported as a calibration plot with its slope and intercept and as the Brier score, and the predicted probabilities are not adjusted afterwards. The support vector machine output is not a probability and is converted into one by Platt scaling.

Performance is reported separately by sex, by age group, by body mass index group and by insomnia severity, taken as the mean of each participant's Insomnia Severity Index scores, which is not used as a predictor.

Two further analyses are planned and are not covered by the sample-size analysis. The first labels each window ending at an administration date with the answer given at that date, so that a participant contributes several observations, and requires that all observations from one participant be kept within a single subset when the data are partitioned. Only data recorded within a window enter the predictors for the label of that window. The second classifies insomnia severity from the Insomnia Severity Index.

The report of the analysis will follow the TRIPOD+AI reporting guideline [[18]](https://www.zotero.org/google-docs/?cUi5Fk).

# 6. Selection Bias and Generalizability

The cohort is recruited from existing Withings device users, while the target population for the detection model is the general population in France. The two differ in the distribution of the collected variables, and what requires adjustment is how well the model performs in the target population. Where distributions of those variables in the target population can be obtained, the cohort can be reweighted to match them by post-stratification or raking, which require population totals alone [[19]](https://www.zotero.org/google-docs/?9njQdm).

Reweighting corrects only the differences it can measure. Two people with the same recorded characteristics, one a participant in this study and one not, may still differ in ways the study does not record, such as technological proficiency or health awareness. If those unrecorded differences affect insomnia, the model trained on the cohort will not perform in the general population as it does in the cohort. The study also cannot establish whether such differences exist, because that would require measuring insomnia status in people who are not participants.

The adjustment applied and the performance obtained with and without it will be reported with the analysis.

# 7. Distribution of Enrolled Participants by Device Ownership

Figure B1: Distribution of enrolled participants across device ownership categories

# 8. Recruitment Procedures

Participant recruitment is planned for a 26-week period and is conducted through the Withings Customer Relationship Management (CRM) database and various social media platforms. The prospective attrition model detailed in Supplementary Materials A projects that about 83,662 users are to be contacted to reach the analysis set, an outreach drawn from the active Withings user base in France.

Targeting through the CRM database identifies registered Withings users who own and have installed at least one of the sleep monitoring devices required for study inclusion. Eligibility for CRM-based contact requires that these users have demonstrated activity, defined as interaction with either one of the specified sleep devices or the WMA, within the 30-day period prior to outreach. Eligible individuals receive a recruitment email that contains an invitation to participate and includes a button designed to redirect the user into the WMA to directly access the digital forms for eligibility verification and informed consent.

We recruit participants by posting content on social media that was designed to spread naturally through user shares and follows, without using any paid advertising. This content is disseminated through posts and temporarily visible content on selected platforms: LinkedIn, Twitter, Instagram, and Facebook. Instagram account owners who are under marketing partnership contracts with Withings may also contribute to recruitment outreach by sharing this prepared content on a voluntary basis, which is consistent with the study's non-commercial research objectives.

A contingency plan involves targeted, paid advertising via Facebook and Instagram utilizing criteria derived from the internal CRM database. This measure is planned to be implemented only if the number of enrolled participants is below 3,000 individuals after the first month of the recruitment period.

The enrollment and consent process is conducted digitally through the WMA. Potential participants first review an electronic information notice, after which they use an integrated digital form to provide informed consent and complete their eligibility verification. Following successful screening and receipt of consent, an automated email confirms the participant's official inclusion in the study and provides a downloadable copy of the information notice for their records.

# References

[1.](https://www.zotero.org/google-docs/?0gwMX7)	[Vapnik VN, Chervonenkis AY. (Translated by B. Seckler).](https://www.zotero.org/google-docs/?0gwMX7)

[2.](https://www.zotero.org/google-docs/?0gwMX7)	[Riley RD, Ensor J, Snell KIE, Harrell FE, Martin GP, Reitsma JB, Moons KGM, Collins G, Van Smeden M. Calculating the sample size required for developing a clinical prediction model. BMJ 2020 Mar 18;m441. doi: 10.1136/bmj.m441](https://www.zotero.org/google-docs/?0gwMX7)

[3.](https://www.zotero.org/google-docs/?0gwMX7)	[Sample Size Requirements for Popular Classification Algorithms in Tabular Clinical Data: Empirical Study - PMC. Available from: https://pmc-ncbi-nlm-nih-gov.proxy.insermbiblio.inist.fr/articles/PMC11688588/ [accessed Aug 22, 2025]](https://www.zotero.org/google-docs/?0gwMX7)

[4.](https://www.zotero.org/google-docs/?0gwMX7)	[Lee L-H, Chen C-H, Chang W-C, Lee P-L, Shyu K-K, Chen M-H, Hsu J-W, Bai Y-M, Su T-P, Tu P-C. Evaluating the performance of machine learning models for automatic diagnosis of patients with schizophrenia based on a single site dataset of 440 participants. Eur Psychiatry 2021 Dec 23;65(1):e1. PMID:34937587](https://www.zotero.org/google-docs/?0gwMX7)

[5.](https://www.zotero.org/google-docs/?0gwMX7)	[Floares AG, Ferisgan M, Onita D, Ciuparu A, Calin GA, Manolache FB. The smallest sample size for the desired diagnosis accuracy. Int J Oncol Cancer Ther 2017;2(13–19):6.](https://www.zotero.org/google-docs/?0gwMX7)

[6.](https://www.zotero.org/google-docs/?0gwMX7)	[van der Ploeg T, Austin PC, Steyerberg EW. Modern modelling techniques are data hungry: a simulation study for predicting dichotomous endpoints. BMC Med Res Methodol 2014 Dec 22;14(1):137. doi: 10.1186/1471-2288-14-137](https://www.zotero.org/google-docs/?0gwMX7)

[7.](https://www.zotero.org/google-docs/?0gwMX7)	[Constantin MA, Schuurman NK, Vermunt JK. A general Monte Carlo method for sample size analysis in the context of network models. Psychol Methods US: American Psychological Association; 2023; doi: 10.1037/met0000555](https://www.zotero.org/google-docs/?0gwMX7)

[8.](https://www.zotero.org/google-docs/?0gwMX7)	[Zimmer F, Debelak R. Simulation-based design optimization for statistical power: Utilizing machine learning. Psychol Methods US: American Psychological Association; 2025;30(3):513–536. doi: 10.1037/met0000611](https://www.zotero.org/google-docs/?0gwMX7)

[9.](https://www.zotero.org/google-docs/?0gwMX7)	[numpy.random.Generator.multivariate_normal — NumPy v2.3 Manual. Available from: https://numpy.org/doc/stable/reference/random/generated/numpy.random.Generator.multivariate_normal.html [accessed Oct 10, 2025]](https://www.zotero.org/google-docs/?0gwMX7)

[10.](https://www.zotero.org/google-docs/?0gwMX7)	[Morin CM, Jarrin DC. Epidemiology of Insomnia: Prevalence, Course, Risk Factors, and Public Health Burden. Sleep Med Clin 2022 June 1;17(2):173–191. doi: 10.1016/j.jsmc.2022.03.003](https://www.zotero.org/google-docs/?0gwMX7)

[11.](https://www.zotero.org/google-docs/?0gwMX7)	[Leger D, others. Prevalence of insomnia in a survey of 12,778 adults in France. J Sleep Res 2000;9(1):35–42.](https://www.zotero.org/google-docs/?0gwMX7)

[12.](https://www.zotero.org/google-docs/?0gwMX7)	[Cheng Y-H, Lech M, Wilkinson RH. Simultaneous Sleep Stage and Sleep Disorder Detection from Multimodal Sensors Using Deep Learning. Sensors 2023;23(7). doi: 10.3390/s23073468](https://www.zotero.org/google-docs/?0gwMX7)

[13.](https://www.zotero.org/google-docs/?0gwMX7)	[Kusmakar S, Karmakar C, Zhu Y, Shelyag S, Drummond SPA, Ellis JG, Angelova M. A machine learning model for multi-night actigraphic detection of chronic insomnia: development and validation of a pre-screening tool. R Soc Open Sci 2021 June;8(6):202264. doi: 10.1098/rsos.202264](https://www.zotero.org/google-docs/?0gwMX7)

[14.](https://www.zotero.org/google-docs/?0gwMX7)	[Hamida ST-B, Ahmed B, Penzel T. A Novel Insomnia Identification Method Based on Hjorth Parameters. 2015 IEEE Int Symp SIGNAL Process Inf Technol ISSPIT 2015. p. 548–552.](https://www.zotero.org/google-docs/?0gwMX7)

[15.](https://www.zotero.org/google-docs/?0gwMX7)	[Sharma M, Dhiman HS, Acharya UR. Automatic identification of insomnia using optimal antisymmetric biorthogonal wavelet filter bank with ECG signals. Comput Biol Med 2021 Apr 1;131:104246. doi: 10.1016/j.compbiomed.2021.104246](https://www.zotero.org/google-docs/?0gwMX7)

[16.](https://www.zotero.org/google-docs/?0gwMX7)	[Shahin M, Ahmed B, Hamida ST-B, Mulaffer FL, Glos M, Penzel T. Deep Learning and Insomnia: Assisting Clinicians With Their Diagnosis. IEEE J Biomed Health Inform 2017 Nov;21(6):1546–1553. doi: 10.1109/JBHI.2017.2650199](https://www.zotero.org/google-docs/?0gwMX7)

[17.](https://www.zotero.org/google-docs/?0gwMX7)	[Josse J, Chen JM, Prost N, Varoquaux G, Scornet E. On the consistency of supervised learning with missing values. Stat Pap 2024 Dec 1;65(9):5447–5479. doi: 10.1007/s00362-024-01550-4](https://www.zotero.org/google-docs/?0gwMX7)

[18.](https://www.zotero.org/google-docs/?0gwMX7)	[Collins GS, Moons KGM, Dhiman P, Riley RD, Beam AL, Calster BV, Ghassemi M, Liu X, Reitsma JB, Smeden M van, Boulesteix A-L, Camaradou JC, Celi LA, Denaxas S, Denniston AK, Glocker B, Golub RM, Harvey H, Heinze G, Hoffman MM, Kengne AP, Lam E, Lee N, Loder EW, Maier-Hein L, Mateen BA, McCradden MD, Oakden-Rayner L, Ordish J, Parnell R, Rose S, Singh K, Wynants L, Logullo P. TRIPOD+AI statement: updated guidance for reporting clinical prediction models that use regression or machine learning methods. BMJ British Medical Journal Publishing Group; 2024 Apr 16;385:e078378. PMID:38626948](https://www.zotero.org/google-docs/?0gwMX7)

[19.](https://www.zotero.org/google-docs/?0gwMX7)	[Elliott MR, Valliant R. Inference for Nonprobability Samples. Stat Sci Institute of Mathematical Statistics; 2017;32(2):249–264.](https://www.zotero.org/google-docs/?0gwMX7)
